# A rare truncal collateral lymph drainage pathway seen on indocyanine green lymphography in patients with secondary lower limb lymphedema

**DOI:** 10.1016/j.jvscit.2023.101126

**Published:** 2023-02-14

**Authors:** Solji Roh, Isao Koshima, Toshiro Mese, Hirofumi Imai, Shuhei Yoshida, Shuji Yamashita

**Affiliations:** a International Center for Lymphedema, Hiroshima University Hospital, Hiroshima, Japan; b Plastic Surgery Department, Kawasaki Medical School Hospital, Okayama, Japan

**Keywords:** Lymphedema, Indocyanine green, Lymphography, Truncal collateral lymphatic pathway, Genital lymphedema

## Abstract

**Objective:**

Although collateral lymphatic vessels are known to develop in patients with lymphedema, little is known about their significance. In this study, we investigated truncal collateral lymphatic drainage pathways in patients with lower limb lymphedema using indocyanine green (ICG) lymphography.

**Methods:**

The ICG fluorescence images and clinical characteristics of 80 consecutive patients (160 lower limbs) with secondary leg lymphedema who underwent ICG lymphography between September 2020 and September 2022 were retrospectively reviewed.

**Results:**

Seven patients were identified to have a truncal collateral lymphatic drainage pathway starting in the lateral abdomen and running in the direction of the ipsilateral axillary lymph nodes. These patients had particularly severe symptoms of lymphedema around the thigh or abdominal region or had genital lymphedema.

**Conclusions:**

A truncal collateral lymphatic drainage pathway may be associated with severe lower limb lymphedema, particularly if involving the genitals.

Understanding the pathophysiology of lymphedema requires knowledge of the lymphatic drainage pathways. Various imaging modalities have been used to visualize the lymphatic vessels, including direct lymphangiography, ultrasound imaging, indocyanine green (ICG) lymphography, lymphoscintigraphy, and computed tomography or magnetic resonance lymphangiography. Since the 1990s, ICG lymphography has become a widespread imaging method because it is easy to perform and can depict superficial lymph flow in real time.^1^ In patients with normal lymphatic function, the superficial lymphatic vessels of the lower limb follow a linear pattern on ICG examination, originating from the medial side of the foot, coursing along the anteromedial side of the lower limb, and heading in the direction of the inguinal lymph nodes.^2^^,^^3^ However, in a patient with lower limb lymphedema, ICG lymphography reveals a tortuous and reticular pattern or diffuse blurred pattern. It is well known that this reticular or diffuse pattern indicates impairment of the lymphatic circulation and is associated with formation of a collateral lymphatic drainage pathway. Nowadays, with the advent of more advanced imaging modalities, lymphatic drainage patterns and structures can be seen in more detail than previously. However, there is still limited information on collateral lymphatic pathways in patients with lymphedema. We believe that better understanding and mapping of variant collateral lymphatic pathways may help to predict the severity of lymphedema and improve therapeutic decision-making in patients with severe intractable lymphedema.

In 1874, Sappey^4^ described a tributary area of the axillary and inguinal lymph nodes. He divided the lymphatic system in the trunk into four regions based on the vertical midline and a theoretical horizontal line drawn around the umbilicus. He claimed that each of these regions drained into the ipsilateral axillary or inguinal nodes and that lymphatic drainage from each region never crossed these lines. This finding led to the rationale for the resection of specific lymph nodes when treating a primary tumor.^5^ Moreover, it was found that collateral drainage pathways can form complementary pathways when there are alterations in lymphatic drainage routes as a result of disruption of the normal lymphatic circulation by lymph node dissection or deterioration of lymphatic vessels.^6^^,^^7^ A more detailed understanding of these collateral lymphatic drainage pathways may help to improve our treatment strategies for patients with intractable lower limb lymphedema.

In this study, we reviewed our clinical experience in patients with lower limb lymphedema and the variant patterns of lymphatic drainage seen on ICG lymphography in severe cases, particularly truncal collateral drainage patterns in the flank region.

## Methods

### Data collection

ICG lymphography images obtained in the International Center for Lymphedema at Hiroshima University Hospital for patients with lower limb lymphedema between September 2020 and September 2022 were retrospectively reviewed. At our center, ICG lymphography is performed routinely in these patients to evaluate the severity of lymphedema and identify the most severely affected regions. Information on patient demographics, etiologies, and clinical characteristics was collected. Detailed information on the history and symptoms of lymphedema was obtained from questionnaires. The study inclusion criteria were an age of >20 years and secondary lower limb lymphedema as a result of surgery for gynecologic or urologic cancer and lymphadenectomy. The following exclusion criteria were applied: a history of surgery in the upper body, especially the trunk region, and a history of treatment for breast cancer.

The study was approved by the institutional review board of Hiroshima University Hospital and conducted in accordance with the Declaration of Helsinki. All patients provided informed consent.

### ICG lymphography

ICG lymphography is performed by injecting ICG solution (0.2 mL of 0.5% Diagnogreen; Daiichi-Sankyo) subcutaneously into the first web spaces of both feet and lateral border of the lateral malleolus. Fluorescent images of the lymphatic drainage pathways are obtained by an infrared camera (Photodynamic Eye; Hamamatsu Photonics K.K.) 1 hour after the injection. The images are recorded in real time by photographs and videos.

### Analysis of ICG lymphography images

ICG lymphography images were classified by the fluorescent pattern (diffuse or linear) and location of the fluorescent region (lower abdomen [umbilicus to pubic tubercle], genital area, thigh, lower leg, or foot) by lymphedema specialists. Linear fluorescent patterns heading in the direction of the inguinal lymph nodes were considered normal, and diffuse blurred patterns were considered to reflect lymphatic drainage obstruction in accordance with previous studies.^8^ A linear lymphatic drainage pathway that arose from the lower abdomen or inguinal region, crossed the theoretical horizontal lymphatic drainage border at the level of the umbilicus, and headed to the axillary region was termed a truncal collateral lymphatic pathway. The direction and number of truncal collateral lymphatic pathways were recorded and analyzed. Eighty patients with lower limb lymphedema were classified into two groups according to whether a truncal collateral lymphatic pathway was identified. The clinical information and ICG lymphography images of the two groups were investigated.

## Results

### Demographic and clinical characteristics

Eighty patients (160 lower limbs) with secondary lower limb lymphedema underwent routine ICG lymphography during the study period. There were no ICG-related complications. The patient characteristics are shown in Table I. The median patient age was 62 years (range, 30-92 years), and the mean body mass index (calculated as kg/m^2^) was 23.6 (range, 16.2-34.5).

**Table I:** Patient demographics and clinical characteristics

	All patients	No, truncal collateral formation	Yes, truncal collateral formation
Number of patients	80	73	7
Sex, No.			
Female	76	69	7
Male	4	4	0
Location of lymphedema, No.			
Bilateral lower limb	68	61	7
Unilateral lower limb	12	12	0
Age, years, median (range)	62 (30-92)	63 (30-86)	52 (38-92)
BMI, kg/m2, average (range)	23.6 (16.2-34.5)	23.8 (16.2-34.5)	22.8 (18.53-29.9)
Etiology, No.			
Cervical cancer	59	53	6
Endometrial cancer	11	11	0
Ovarian cancer	6	5	1
Prostate cancer	2	2	0
Testicular cancer	2	2	0
Adjuvant chemoradiation therapy, No.			
Yes	65	60	5
No	15	13	2
Average duration of lymphedema, years (range)	8.5 (1-31)	7.9 (1-24)	12.0 (1-31)
ISL lymphedema stage, No.			
Stage 0	0	0	0
Stage Ⅰ	0	0	0
Stage Ⅱ	0	0	0
Stage late Ⅱ	35	33	2
Stage Ⅲ	45	40	5

*BMI*, Body mass index; *ISL*, International Society of Lymphedema.

The etiology of the secondary lymphedema was cervical, endometrial, ovarian, prostate, or testicular cancer. Most of the patients (65 of 80, 68.8%) underwent tumor resection, lymphadenectomy, and adjuvant chemoradiotherapy. The patients were classified clinically as having late stage Ⅱ (35 of 80, 43.8%) or stage Ⅲ (45 of 80, 56.2%) lymphedema according to the International Society of Lymphedema stage criteria.

### Patients with a truncal collateral lymphatic pathway

On analysis of ICG lymphography images, 7 of the 80 patients were found to have a truncal collateral pathway, with lymphatic drainage crossing the watershed area in the abdomen toward the ipsilateral axillary lymph nodes (Table II). This section summarizes three representative cases.

**Table II:** Characteristics of patients with a truncal collateral lymphatic pathway

	Case 1	Case 2	Case 3	Case 4	Case 5	Case 6	Case 7
Sex	F	F	F	F	F	F	F
Age, years	56	42	92	68	43	38	52
Cancer type	Cervical cancer	Cervical cancer	Cervical cancer	Ovary cancer	Ovary cancer	Cervical cancer	Cervical cancer
LN dissection (Yes/No)	Yes	Yes	Yes	Yes	Yes	Yes	Yes
Adjuvant chemoradiotherapy (Yes/No)	Yes	Yes	Yes	Yes	Yes	No	No
Leg edema	Bilateral	Bilateral	Bilateral	Bilateral	Bilateral	Unilateral	Unilateral
ISL stage	Stage Ⅲ	Stage Ⅲ	Stage Ⅲ	Stage Ⅲ	Stage Ⅲ	Stage late Ⅱ	Stage late Ⅱ
Postoperation periods, years	16	14	41	2	6	5	14
Edema duration, years	16	14	31	1	4	4	14
Region edema first appeared	Thigh	Genital region	Genital region	Thigh	Thigh	Thigh	Thigh
Region edema most severe	Thigh	Genital region	Genital region	Thigh	Lower abdomen	Thigh	Thigh
Symptom associated with genital lymphedema							
ICG diffuse pattern on the genital region (Yes/No)	No	Yes	Yes	Yes	Yes	Yes	Yes
Edema of the mons pubis (Yes/No)	Yes	Yes	Yes	Yes	Yes	Yes	Yes
Papillomatosis of the labia (Yes/No)	No	Yes	Yes	Yes	Yes	Yes	No
Lymphorrhea of the labia (Yes/No)	No	Yes	Yes	Yes	No	No	No

*ICG*, Indocyanine green; *ISL*, International Society of Lymphedema; *LN*, lymph node.

#### Case 1

**Fig 1:**
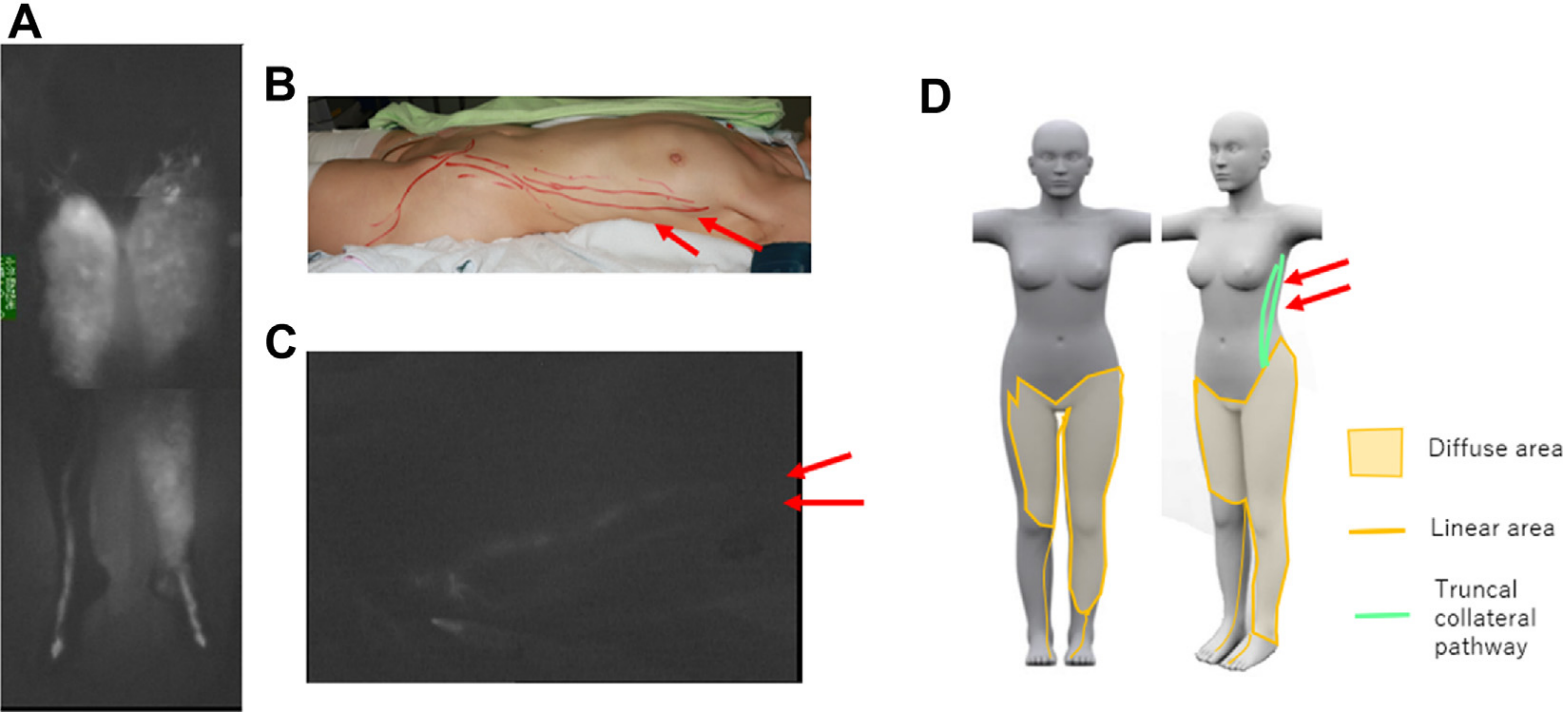
Case 1: **(A)** indocyanine green (ICG) image of the leg, **(B)** truncal collateral pathways (marked with a red pen), **(C)** two truncal collateral pathways on ICG, and **(D)** ICG image illustration and truncal collateral pathways.

The patient was a 56-year-old woman with bilateral secondary lower limb lymphedema. At the age of 40 years, she had undergone treatment for cervical cancer, including radical hysterectomy, pelvic lymph node dissection, and radiotherapy. Her lymphedema had started in the left thigh at the age of 50 years. She underwent bilateral lower limb lymphaticovenous anastomosis surgery at the age of 55 years and again at 56 years. ICG lymphography revealed a diffuse pattern in both thighs and in the left leg (Fig 1, *A*) and two collateral pathways extending from the left inguinal lesion to the mid axillary region (Fig 1, *B-D*).

#### Case 2

**Fig 2:**
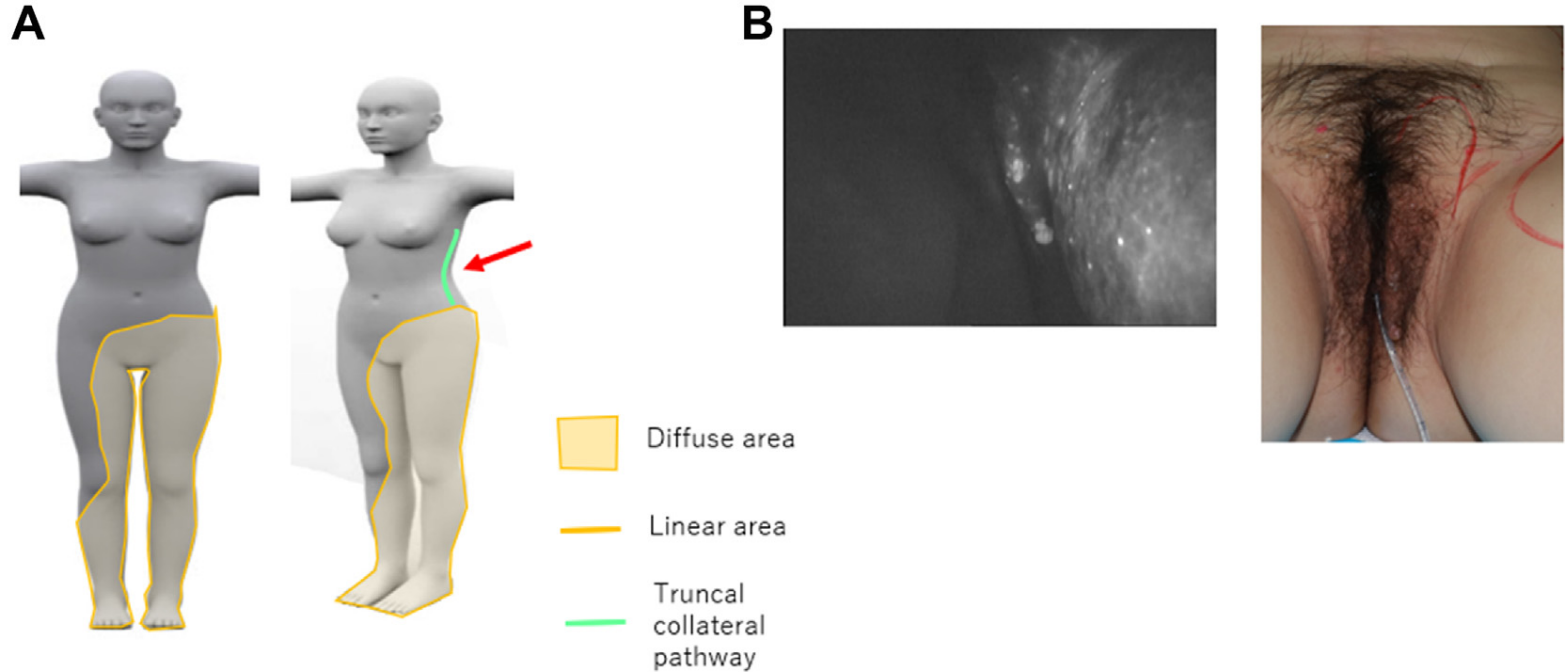
Case 2: **(A)** indocyanine green (ICG) image illustration and truncal collateral pathway and **(B)** ICG image of labia majora papillomatosis.

The patient was a 42-year-old woman who was diagnosed with secondary bilateral lower limb lymphedema after surgery for cervical cancer. Fourteen years earlier, she had undergone radical hysterectomy, pelvic lymph node dissection, and radiotherapy to the pelvic and vaginal areas. Her edema had started in the genital region immediately after the resection of the tumor and gradually spread to involve the entire left leg and right thigh. Swelling of the labia majora and genital papillomatosis had appeared 9 years earlier. She also had lymph leakage from genital papillomatosis, for which she was wearing incontinence pads on most days. Her ICG lymphography examination revealed a truncal collateral lymphatic pathway along the left midaxillary line (Fig 2, *A* and *B*).

#### Case 3

**Fig 3:**
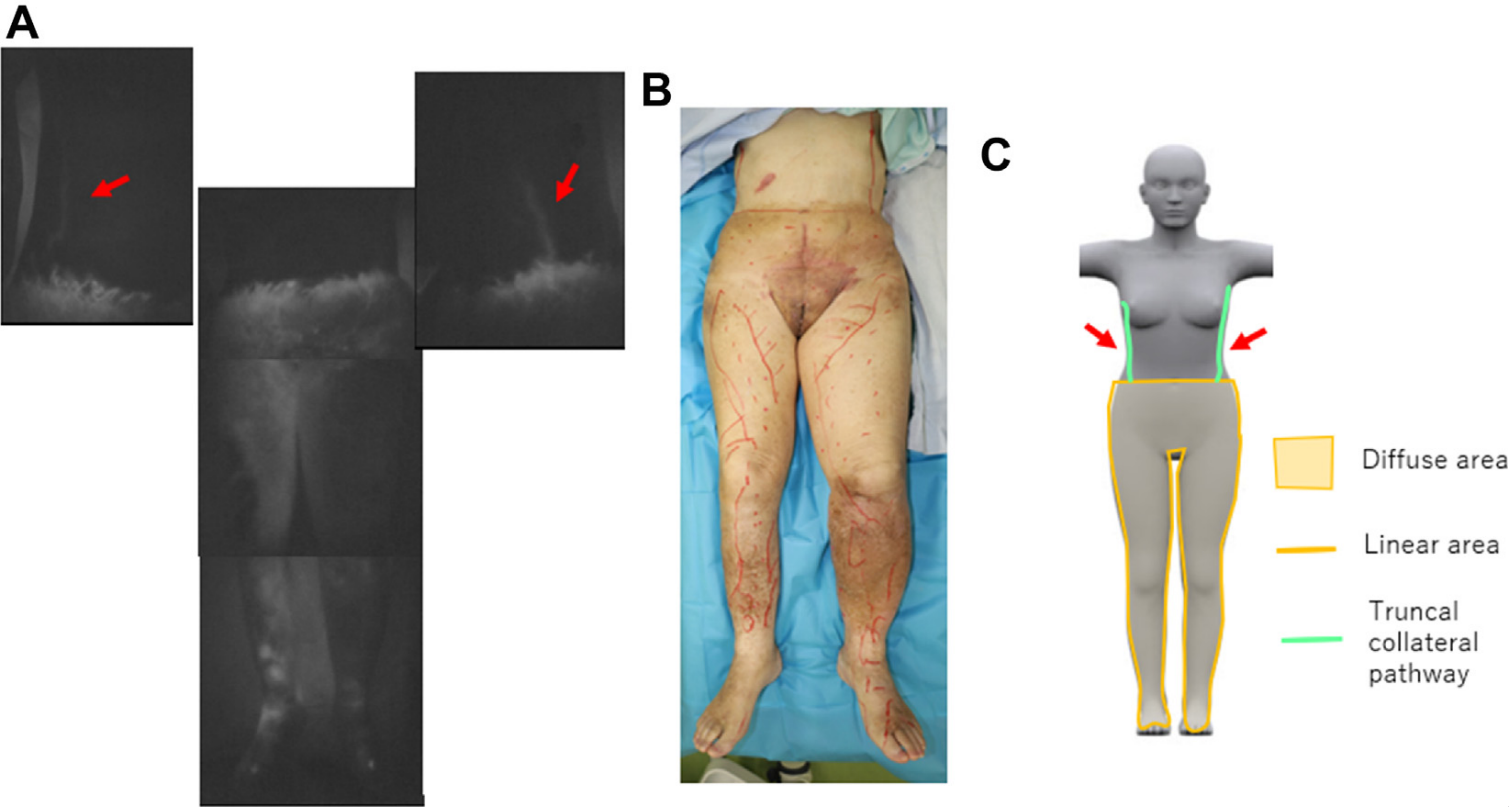
Case 3: **(A)** indocyanine green (ICG) image of the lower limb and bilateral truncal collateral pathways, **(B)** general appearance of the lower limb, and **(C)** ICG image illustration and bilateral truncal collateral pathways.

The patient was a 92-year-old woman with bilateral secondary lower limb lymphedema after cervical cancer surgery. Forty-one years earlier, she had undergone radical hysterectomy, pelvic lymph node dissection, and radiotherapy to the pelvic and vaginal areas. Ten years after the surgery, edema of the mons pubis had appeared and gradually extended to both thighs. She developed edematous changes in the labia majora, vestibular papillomatosis, and lymphorrhea. Her ICG examination showed a diffuse dermal backflow pattern from both feet to the abdominal region and bilateral truncal collateral lymphatic pathways. These collateral pathways arose from the lower abdomen and extended to the anterior axillary region on one side and to the mid axillary region on the other (Fig 3, *A-C*).

### Comparison of patients according to whether a collateral lymphatic pathway was present

The ICG lymphography images of 80 patients were analyzed by lymphedema specialists. The ICG images were classified by the location of the fluorescent region (lower abdomen [umbilicus to pubic tubercle], genital area, thigh, lower leg, or foot) and pattern (linear or diffuse). According to whether a truncal collateral lymphatic pathway was present, the location of the diffuse ICG pattern was investigated (Table III). A diffuse ICG pattern in the lower abdomen, genital area, or thigh regions was more common in the seven patients with a collateral lymphatic pathway. Genital lymphedema associated with symptoms, such as edema of the mons pubis or labial area, papillomatosis, discomfort, or lymphorrhea, was also more common in these seven patients.

**Table III:** Comparison of patients according to whether a collateral lymphatic pathway was present

	No, truncal collateral pathway (n = 73)	Yes, truncal collateral pathway (n = 7)
ICG diffuse pattern region, No.		
Lower abdomen	10	6
Genital region	19	6
Both thigh	52	7
Both lower leg	46	5
Both feet	8	2
Genital lymphedema associated symptom, No.		
Yes	38	7
No	35	0

*ICG*, Indocyanine green.

## Discussion

In this study, we analyzed ICG lymphography images from 80 patients with secondary lower limb lymphedema after the resection of a malignant tumor with lymph node dissection. Seven of these patients were identified to have a truncal collateral lymphatic pathway.

In all seven cases, the unusual lymphatic pathway crossed the horizontal line at the level of the umbilicus, which is the arbitrary anatomical border or watershed lymphatic drainage area between the upper and lower body described by Sappey.^4^ These collateral pathways emerged from the lateral abdomen and traveled to the ipsilateral anterior or mid axillary region.

In 2013, Suami et al^7^ established an animal model for investigating postoperative alterations in the lymphatic drainage pathway. In 2016, two of the investigators in the present paper reported truncal collateral lymphatic pathways in 3 of 96 patients with lower extremity lymphedema.^9^ In all three cases, the collateral pathway arose from the abdomen and traveled in the direction of the ipsilateral axillary or clavicular lymph nodes. These pathways were similar in location and direction to those found in the present study. To the best of our knowledge, there are no other published reports on changes in lymphatic pathways seen on ICG lymphography after lymph node dissection in humans, possibly because of their rarity and unpredictability.

Although collateral pathways were demonstrated in the 1970s and 1980s by direct lymphangiography,^10^ they received little attention because of the declining use of direct lymphangiography. When performing direct lymphangiography, contrast material such as lipiodol is usually injected into the dermis, where it is absorbed by the initial lymphatics and fills the lymphatic vessels; however, lipiodol was found to cause inflammation or lymphatic vessel blockage because of its oily properties, resulting in obliteration of the lymphatic vessels.^11^ Therefore, direct lymphangiography was eventually superseded by other imaging methods, such as ICG lymphography, ^99m^Tc radionuclide lymphoscintigraphy, and magnetic resonance lymphangiography.

In 1971, Escobar-Prieto et al^12^ examined 220 patients by direct lymphangiography and found that when lymphatic flow was blocked, an alternative backflow or collateral circulation was formed to overcome the obstruction. They concluded that collateral pathways could develop in three ways, namely, lymphaticolymphatic anastomosis, lymphaticovenous communication, and a para-lymphatic pathway that could include accumulation in the interstitial space or flow in the perivascular and perineural spaces.

Bruna^13^^,^^14^ analyzed images of lower extremities obtained by direct lymphangiography and divided those with a collateral lymphatic circulation into a typical pattern (cutaneous, subcutaneous, parietal, and perivisceral) and an atypical pattern (lymphovenous communications, extravasation into cavities, and internal lymphatic fistula).

We believe that our findings strongly correlate with the knowledge gleaned from direct lymphangiography. We also hypothesized that a truncal collateral pathway could develop in patients with progressive lymphedema in the lower abdomen or thigh. For example, in our study, all seven patients found to have a truncal collateral pathway complained of subjective symptoms of lymphedema, such as feelings of heaviness, tension, and swelling in the lower abdominal or upper thigh region. Objective signs, such as firmness, tightness, or discoloration, were observed mainly in the same regions. Furthermore, many of these patients also had signs of genital lymphedema, such as lymphorrhea, severe swelling of the labia majora, and papillomatosis. ICG examination in these patients usually revealed a blurred dermal backflow pattern in the thigh or pelvic region. Therefore, development of a truncal collateral pathway can be considered to reflect very severe secondary lymphedema of the thigh or abdominal region. Given that symptoms of secondary lower limb lymphedema after surgery and lymph node dissection for gynecologic cancer usually start from the inguinal or thigh region, it is probable that the lymphatic vessels around an inguinal lesion are most damaged. Moreover, considering that a collateral lymphatic pathway is usually formed in a complementary manner to maintain lymphatic drainage in a severely obstructed lymphatic circulation, a truncal collateral pathway may help to identify a solution for improving lymphatic drainage in patients with intractable lymphedema. For example, we hypothesize that the collateral pathway should be preserved to provide an alternative route for lymphatic drainage. By surgically bridging a lymph drainage-obstructed area and a patent area with normal lymph flow, lymphatic drainage may be restored from the affected lower limb to the patent upper limb. Further developments in surgical techniques could allow bridging between the inguinal and axillary lymph systems, thereby allowing blocked lymph flow in the lower limb to flow upward and reach the axillary or cervical lymphatic system.

In 1935, Gillies and Fraser^15^ designed an operation that facilitated flow of lymph from the lower limb to the axillary region by transplanting skin and subcutaneous tissue from the arm to the trunk, which nowadays corresponds to a pedicled arm flap. In 1982, Clodius et al^16^ treated patients with unilateral lower limb lymphedema by using a pedicled groin flap to drain lymph from the affected leg to the normal leg. Further evaluation of the effect of this method, which involves connection of two distant lymphatic systems, on improving the lymphatic drainage may be needed.

Nonetheless, these empirical results must be interpreted with caution, and a number of limitations should be borne in mind. First, although this study demonstrates some rare but significant findings that link the lymphatic system of the leg with that of the upper body, we are unable to perform a statistical analysis or elucidate a biophysical mechanism in detail. Outstanding questions include how long the collateral pathways last; whether they develop via a specific pathway, as with tumor metastasis; and how many pathways are formed. Larger-scale, longer-term studies are needed to determine its implied meaning and apply it to the treatment of intractable lymphedema. Second, only limited data could be acquired owing to the low spatial resolution of ICG lymphography. More comprehensive examination using multiple image modalities, such as magnetic resonance lymphangiography, computed tomography lymphangiography, or photoacoustic lymphangiography, is needed to reveal the structures and patterns of truncal collateral lymphatic vessels and to interpret the changes in lymphatic structures seen in patients with lymphedema in more detail. Finally, further research on genital lymphedema is needed to evaluate its true impact on the development of a truncal collateral lymphatic pathway.

Changes in the lymphatic drainage pathways and formation of collateral pathways in patients with lymphedema have received little attention until now. Further investigation may shed light on the pathophysiology of lymphedema.

## Conclusions

In order to fully understand lymphedema, it is necessary to know the normal lymphatic drainage pathway as well as its variants. Truncal collateral lymphatic pathways are rare but are associated with severe secondary lower limb lymphedema, particularly when involving the genital area.
